# Anti-oxidative stress response genes: bioinformatic analysis of their expression and relevance in multiple cancers

**DOI:** 10.18632/oncotarget.1658

**Published:** 2013-12-15

**Authors:** Barak Rotblat, Thomas G. P. Grunewald, Gabriel Leprivier, Gerry Melino, Richard A. Knight

**Affiliations:** ^1^ Medical Research Council, Toxicology Unit, Leicester University, Leicester, UK; ^2^ INSERM Unit 830 “Genetics and Biology of Cancer”, Institut Curie Research Center, Paris, France; ^3^ Department of Molecular Oncology, British Columbia Cancer Research Centre and Department of Pathology, University of British Columbia, Vancouver, BC, Canada; ^4^ Department of Experimental Medicine and Surgery, Biochemistry IDI-IRCCS Laboratory, University of Rom ‘Tor Vergata’, Rome, Italy

**Keywords:** anti-oxidant genes, glutathione, thioredoxin, breast cancer, lung cancer, NRF2, G6PD

## Abstract

Cells mount a transcriptional anti-oxidative stress (AOS) response program to scavenge reactive oxygen species (ROS) that arise from chemical, physical, and metabolic challenges. This protective program has been shown to reduce carcinogenesis triggered by chemical and physical insults. However, it is also hijacked by established cancers to thrive and proliferate within the hostile tumor microenvironment and to gain resistance against chemo- and radiotherapies. Therefore, targeting the AOS response proteins that are exploited by cancer cells is an attractive therapeutic strategy. In order to identify the AOS genes that are suspected to support cancer progression and resistance, we analyzed the expression patterns of 285 genes annotated for being involved in oxidative stress in 994 tumors and 353 normal tissues. Thereby we identified a signature of 116 genes that are highly overexpressed in multiple cancers while being only minimally expressed in normal tissues. To establish which of these genes are more likely to functionally drive cancer resistance and progression, we further identified those whose overexpression correlates with negative patient outcome in breast and lung carcinoma. Gene-set enrichment, gene ontology, network, and pathway analyses revealed that members of the thioredoxin and glutathione pathways are prominent components of this oncogenic signature and that activation of these pathways is common feature of many cancer entities. Interestingly, a large fraction of these AOS genes are downstream targets of the transcription factors NRF2, NF-kappaB, and FOXM1, and rely on NADPH for their enzymatic activities highlighting promising drug targets. We discuss these findings and propose therapeutic strategies that may be applied to overcome cancer resistance.

## INTRODUCTION

The stressful biological conditions that exist within the tumor microenvironment exert strong adaptive pressure on cancer cells which in turn exploit endogenous pathways to reprogram their transcriptome, proteome, and metabolism to survive and thrive under these conditions [1-6]. Therefore, proteins that facilitate these adaptation processes are attractive drug targets as they are expected to be active only in tumor tissues, which are exposed to stress, but not in non-stressed normal tissues [2, 7, 8]. Oxidative stress is commonly associated with cancer and cancer cells have been shown to promote expression of ROS scavenging pathways in order to survive, proliferate, and resist radio- and chemotherapy [9-11]. While these basic biological principles have been extensively demonstrated and reviewed elsewhere [12], especially in the context of the transcription factor nuclear factor (erythroid-derived 2)-like 2 (NRF2 or NFE2L2) [13-15], it is still not clear which groups of AOS genes are overexpressed in multiple cancers compared with normal tissues. Similarly, it is as yet not defined which groups of AOS genes predict for bad prognosis and in different cancer entities.

Here, we systematically evaluated the mRNA expression patterns of all genes (n=285) annotated by GO(Gene Ontology) as being involved in ‘oxidative stress’ (including AOS genes) in publicly available microarray data sets and identified a sub-group of genes that is highly overexpressed in multiple cancers compared to normal tissues. Subsequently, by using multiple unsupervised analyses, we found that the glutathione and thioredoxin pathways are significantly enriched among these genes. Interestingly, high expression of a significant number of these genes is negatively correlated with survival in breast and lung carcinoma, suggesting that they might play a protective role in cancer cells as opposed to merely reflecting a transcriptional response to oxidative stress. We discuss these genes, the regulators of their expression, their specific role in cancer, and possible therapeutic strategies that can hit these targets.

### Identification of oxidative stress response genes highly expressed in multiple cancers: enrichment in glutathione and thioredoxin pathways-related genes

**Figure 1:**
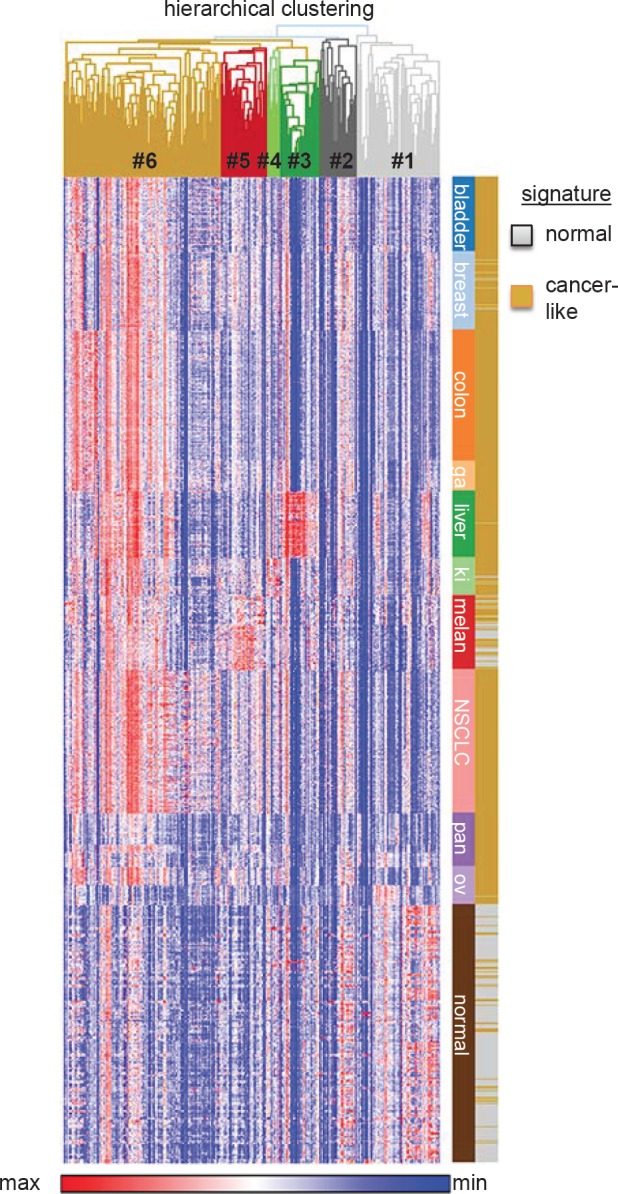
Gene expression patterns of 285 oxidative stress genes in 353 normal tissues and various carcinomas (total n=994, 10 different entities) Gene expression data were retrieved from the Gene Expression Omnibus (GEO; http://www.ncbi.nlm.nih.gov/gds) of published microarray studies (all Affymetrix HG-U133plus2.0). Normal tissue n=353 (GSE3526) [133]. Carcinomas: bladder n=102 (GSE31684, GSE7476), breast n=107 (GSE36774), colorectal n=177 (GSE17536), gastric cancer (ga) n=43 (GSE22377), liver (hepatocellular carcinoma) n=91 (GSE9843), kidney (ki) n=52 (GSE11151), melanoma (melan) n=101 (GSE10282, GSE15605), lung (non-small-cell lung cancer, NSCLC) n=196 (GSE37745), pancreas (pan) n=52 (GSE17891, GSE32676), ovary (ov) n=73 (GSE14001, GSE18520). All microarray data were normalized simultaneously by RMA [134] using custom brainarray (v15.0) ENTREZG CDF-files as previously described [132, 135, 136]. Hierarchical clustering of genes (1-Pearson correlation) and k-means clustering (2 signatures, 10,000 iterations) of microarray samples were performed with GENE-E software (http://www.broadinstitute.org/cancer/software/GENE-E/index.html). Gene expression data were log2 transformed for depiction in a heat-map.

We wondered whether specific oxidative stress response genes are highly overexpressed in cancer as compared to normal tissues. Because we are primarily interested in how cancer cells adapt to their microenvironment found within solid tumors we focused our analysis on carcinomas as they constitute the most frequent type of solid tumors. Using hierarchical clustering, we observed that the 285 genes cluster into 6 clusters (hereafter referred to as “groups”) (Figure 1; Table S1). Groups 2-5 were found to be cancer type specific (Figure 1; Table S1). While these may be interesting in the context of the corresponding cancers entity, they may also reflect genes highly expressed in the tissue of origin, and therefore will require further in-depth analysis. More interestingly, we identified a group of genes that is highly overexpressed in multiple cancers compared to normal tissues (group 6), as well as a group that is highly overexpressed in normal tissues compared to cancers (group 1).

GO analysis using bioprofiling.de [16] (Table S2) confirmed that in both groups ‘response to oxidative stress’ and ‘response to hydrogen peroxide’ were the top two categories (p<10-15) confirming, as expected, that both lists (groups 1 and 6) are significantly enriched in genes involved in oxidative stress response. While the first two GO categories were similar between group 1 and group 6 the third was different. ‘Aging’ was the third identified GO category in the list of genes that are highly expressed in normal tissues (group 1) (p<10-7) while ‘cellular response to hydrogen peroxide’ (p<10-12) was the third category found in the list of genes that are highly expressed in cancer (group 6) (Table S2). It is interesting to note that the expression of AOS genes that are linked to aging is a feature of normal tissue in light of the discussion on the similarities and differences between expression of stress genes in cancer and aging [10, 17].

**Figure 2:**
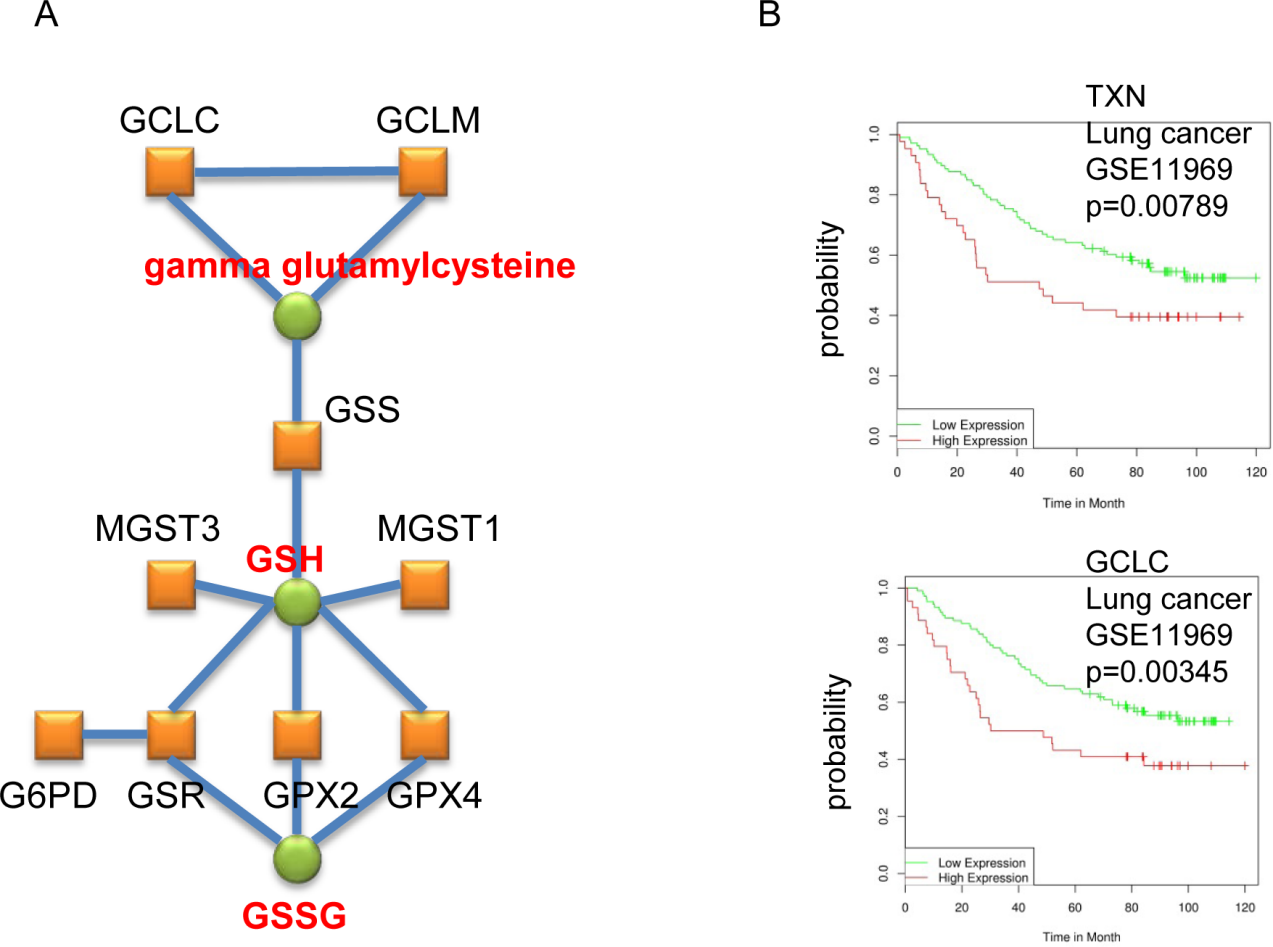
Enrichment of genes coding for enzymes involved in glutathione synthesis in the cancer AOS genes signature A. The depicted gene network was identified by R_SPIDER as statistically enriched in the list of genes that are highly expressed in cancers (group 6) (Table S1). Genes are represented by red boxes, known interactions between the corresponding proteins are displayed as blue lines and metabolites by green circles. B. Typical Kaplan-Meier plots are shown.

To identify possible common biological features of the genes represented in each of the two lists we next queried common protein folds of the encoded proteins. Using Interpro (bioprofiling.de; [16]) we found that the list of genes that are highly expressed in cancer (group 6) is significantly enriched (p<10-6; Table S3) in proteins that contain ‘Alkyl hydroperoxide reductase subunit C/ Thiol specific antioxidant’ domains, ‘Thioredoxin fold’, and ‘Thioredoxin like fold’, whereas the genes that are highly expressed in normal tissues (group 1) did not result in specifically enriched protein fold(s). Moreover, using pathway and network analysis (bioprofiling.de R_Spider; [18]), we found the ‘Glutathione metabolism’ pathway among the genes highly expressed in cancers (11 genes; p=0.01) with a specific sub-group of 9 genes (p<0.005) whose products are known to interact with one another, such as glutamate cysteine ligase catalytic subunit (GCLC) and glutamate cysteine ligase modifier subunit (GCLM) (Figure 2A)[19]. Collectively, these analyses suggest that elevated glutathione synthesis and thioredoxin pathway activity are common features of cancer cells.

### Identification of AOS response genes highly expressed in cancers which predict negative patient outcome

In order to identify possible drug targets within the list of genes that are up-regulated in cancer (group 6) we used the bioprofiling.de GENE_SRV tool that screens a list of genes against publicly available expression and patient survival data [20]. Specifically, this tool identifies cancer entities in which a particular gene signature is significantly enriched for predictors of patient outcome. We found significant predictive power of some genes in group 6 (highly expressed in cancers) in breast and lung cancers (p=0.035) and in chronic lymphocytic leukemia (CLL) (p=0.037). Since this study exclusively addresses AOS genes in solid tumors, we focus our discussion on the first two cancer entities. Kaplan-Meier plots for all genes that exhibited significant predictive power are summarized in Table 1 and Figure S1-3 (typical plots are shown in Figure 2B).

**Table 1:** List of AOS response genes highly expressed in cancers which correlate with outcome in breast or lung cancer The cancer AOS response signature was analyzed using bioprofiling.de GENE_SRV to identify cancers in which these genes have significant predictive power. Only genes that were found to correlate with survival are shown. Gene name, ENTREZ ID, microarray probeset ID and p value are provided. Kaplan-Meier plots for all the indicated genes are displayed in Figure S1-S3.

Breast cancer		ENTREZ ID	
	Gene	(probe ID)	P-value
Bad prognosis	BTG3	10950 (360504)	0.00357
	CASP3	836 (540397)	0.0000453
	CDC2	983 (5360092)	0.0000105
	ECT2	1894 (5420064)	0.00012
	EGLN1	54583 (6130168)	0.00586
	FOXM1	2305 (5390044)	2.51E-08
	G6PD*	2539 (5700072)	0.00748
	GAPDH	2597 (1940184)	0.00321
	HMOX1*	3162 (6180100)	0.000294
	LONP1	9361 (870538)	0.0031
	NUDT1	4521 (6180369)	0.0016
	PRDX4*	10549 (940131)	0.00276
	PSMB5	5693 (3610041)	0.00337
	SELS	55829 (7100450)	0.00844
	SERPINE1	5054 (6840139)	0.00167
	SRXN1*	140809 (3190176)	0.00336
	TXNRD1*	7296 (6220603)	0.00000169

Good prognosis	PON2	5445 (7040022)	0.00457
	SIRT1	23411 (6940021)	0.00918

Lung cancer		NCBI ID	
	NCBI ID	(probe ID)	P-value
Bad prognosis	COL1A1	1277 (926)	0.000675
	GAPDH	2597 (1738)	0.00185
	GCLC*	2729 (14771)	0.00354
	GSS*	2937 (267)	0.00934
	NQO1*	1728 (20812)	0.0045
	RNF7	9616 (12099)	0.00439
	STK24	8428 (10957)	0.00195
	TXN*	7295 (10753)	0.00789
	TXNRD1*	7296 (8394)	0.00284

Good prognosis	NFKB1	4790 (3750)	0.000849


* NRF2 targets

In lung cancer, 9 genes correlated with poor prognosis, including *GCLC*, *NAD(P)H dehydrogenase (quinone) 1 (NQO1)* and *thioredoxin* (TXN), and 1 with good outcome (Figure 2B and Figure S1). In breast cancer, 17 genes were associated with poor prognosis, such as *glucose-6-phosphate dehydrogenase* (*G6PD*), *heme oxygenase* (*decycling*) *1* (*HMOX1*) and *thioredoxin reductase 1* (*TXNRD1*), and only 2 with good outcome (Table 1 and Figure S2-S3). The finding that the majority of the genes are predictors for negative patient outcome supports the model that the AOS response genes, which are up-regulated in cancer, may facilitate cancer cell adaptation to the tumor environment and/or resistance to therapy. We therefore argue that the genes identified by our analyses as being highly overexpressed in carcinomas and correlating negatively with prognosis may constitute attractive drug targets as well, which will be further discussed below.

### Relevance of the glutathione and thioredoxin pathways as essential components of multiple cancers and potential drug targets

#### Thioredoxin pathway

**Figure 3:**
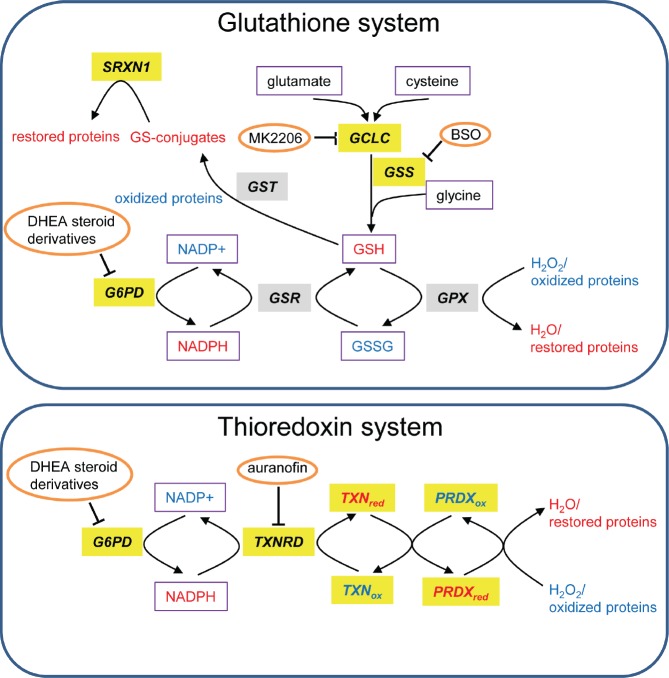
Glutathione and TXN systems Genes that are highly expressed in tumors versus normal tissues are highlighted in gray and those associated with bad prognosis in lung or breast cancer are highlighted in yellow. The redox state of proteins and metabolites is depicted in color (red=reduced and blue=oxidized). Metabolites are boxed and inhibitors are circled. This scheme is adapted from [137].

The thioredoxin system is highly conserved throughout evolution and we observed that multiple members of this system are highly overexpressed in multiple cancers (Figure 1; group 6) and confer dismal prognosis in lung and breast cancers (Table 1 and Figure S1-S3). TXN is a small protein that reduces oxidized proteins and supports peroxiredoxin (PRDX)-mediated H2O2 clearance (Figure 3) [21]. It also positively regulates the activity of PTP1B, the phosphatase of the tyrosine kinase PDGF-beta, leading to increased PDGF-beta signaling [20] and it negatively regulates the tumor suppressor PTEN [22, 23]. These functions point to an oncogenic role of TXN.

In support to this notion, the expression of a number of TXN-related genes has been reported to predict negative patient outcome in multiple cancers [24]. Among the TXN-related genes we identified to be up-regulated in cancers (group 6), TXN expression was associated with reduced survival in various cancers, such as gastric, colorectal, non-small cell lung cancers and squamous cell carcinoma [25-27], whereas TXNRD1 expression was correlated with poor survival in breast cancer and squamous cell carcinoma [28, 29]. Furthermore, PRDX1 level was found to predict poor patient survival in non-small cell lung, ovarian, and breast cancers [30-32], and PRDX3 and PRDX4 expression were correlated with poor prognosis in hepatocellular carcinoma and squamous cell carcinoma respectively [33, 34].

Among TXN pathway inhibitors, the TXN inhibitor PX-12 was shown to be well-tolerated in phase I trials [35]. However, in a phase II trial it exhibited limited therapeutic benefits possibly related to its pharmacokinetics [36], which prompted the development of better TNX inhibitors. An alternative strategy to inhibit TXN is to block TXN reductases [37], such as TXNRD1, that reduce and recycle TXN (Figure 3). TXNRD1 is an interesting drug target as its gene was found in our analysis to be up-regulated in cancers compared with normal tissues (Figure 1A; group 6). Moreover its high expression correlates with worse prognosis in both lung and breast cancer (Table 1 and Figure S1-S2). Indeed, its inhibitor Auranofin [38] can induce apoptosis and inhibit cancer cell growth *in vitro*, and is currently tested in clinical trials for CLL (phase II). Moreover, Auranofin was suggested to be used for treatment of glioblastoma [39], breast [40], lung [41-43], and other cancers [44]. Interestingly, Auranofin is an example of drug repurposing as it is a well-tolerated FDA-approved drug being already used for treatment of rheumatoid arthritis [38].

#### Glutathione pathway

Glutathione is the most abundant antioxidant in the cell and is involved in resistance of cancer cells to oxidative stress arising from detachment, hypoxia, radio- and chemotherapy [45-52]. GCLC and glutathione synthetase (GSS), whose genes were identified by our analysis to be highly overexpressed in cancers (Figure 2) and to confer bad prognosis in patients (Figure S1), are both essential enzymes catalyzing the synthesis of glutathione from glutamate, cysteine and glycine (Figure 3).

Previous reports have highlighted the clinical relevance for some of the glutathione-related genes we identified to be up-regulated in melanoma only (group 5) and in all cancers (group 6). The importance for glutathione S-transferase pi 1 (GSTP1) expression as a factor of bad prognosis and of poor response to chemotherapy has been reported in head and neck, gastric, colon, breast and ovarian cancers [53-60]. In addition, high GCLC and GCLM levels were associated with poor progression-free survival in diffuse large B-cell lymphoma [61], and glutathione peroxidase (GPX) activity was found to be specifically high in prostate and lung cancers compared to corresponding normal tissues [62, 63].

The glutathione pathway can be inhibited using specific drugs such as buthionine sulfoximine (BSO). The latter is a well-known inhibitor of GCLC [64] and has been shown to have only little adverse effects in humans [65, 66]. However, its efficacy as an anticancer drug is limited possibly due to bypass effects by other detoxification pathways such as the TXN pathway. In line with this notion, it was recently demonstrated that only when both the glutathione and the TXN pathways were inhibited simultaneously, using BSO and Auranofin, respectively, there was significant inhibition of head and neck squamous cell carcinoma growth *in vitro* and *in vivo* [67]. The synergistic effects were efficiently blocked by N-acetyl cysteine (NAC), that replenishes glutathione, but not by catalase suggesting that the simultaneous inhibition of TXN and the glutathione pathways rather than redcution of total anti-oxidant cellular capacity is responsible for the growth inhibitory effect [67]. Similarly, it was shown that simultaneous inhibition of TXN and glutathione systems resulted in synergistic killing of lung cancer cells [41]. This was demonstrated using Auranofin and the AKT inhibitor MK2206, whose efficacy depends on the activity of KEAP1. KEAP1 is a known inhibitor of the transcription factor NRF2 that promotes the expression GCLC and other key enzymes in the glutathione synthesis pathway [68-71]. These data once more underscore that there is a synergistic effect caused by simultaneous block of the TXN system and the glutathione pathway. Our finding that genes enriched for both pathways are highly overexpressed in multiple cancers further supports this strategy of inhibiting both pathways simultaneously to achieve effective targeted anti-cancer therapy.

### Transcription factors regulating the cancer AOS response genes and their clinical relevance

#### NRF2

Our first analysis is based on gene expression data that reflects the sum activities of regulators of gene expression including those of transcription factors. We observed that in the genes list that predict poor outcome, 9 are known NRF2 targets (Table 1 and Figure S1-S3). These include genes involved in glutathione and TXN pathways, G6PD that is involved in NADPH generation (Figure 2) and *NQO1* and *HMOX1* that encode detoxification enzymes [68, 69, 72-79]. Because NRF2 promotes the expression of oxidative stress detoxifying proteins, it is not surprising that NRF2 depletion results in increased tumor formation in mice challenged with carcinogens [80-83]. However, cancer cells also exploit NRF2 to reduce oxidative stress and resist chemotherapy [84-87]. In line with these two seemingly opposing NRF2 functions, recent data provides evidence that NRF2 knockout mice develop more K-RAS induced tumors on the one hand, but these are less aggressive on the other hand [88]. These observations support the concept that cancer cells exploit NRF2 to adapt to oxidative stress and to resist chemotherapy. This concept gained support by identification of somatic mutations in *NRF2* itself and in its inhibitor, *KEAP1*, that lead to increased NRF2 activity in tumors (reviewed [13, 84, 87, 89, 90]). It is therefore an attractive strategy to block NRF2 in order to reduce the expression of its downstream target genes that are involved in both the glutathione and TXN pathways. Interestingly, the natural compound Brustatol was recently found to inhibit NRF2 in cells and to promote tumor sensitization to chemotherapy *in vivo* [91], suggesting that NRF2 is druggable and that using an NRF2 antagonist may be a feasible therapeutic strategy.

#### FOXM1

Another transcription factor we found to be deregulated in multiple cancers is FOXM1, an oncogenic protein known to control proliferation, DNA damage repair, angiogenesis, and AOS response [92, 93]. Indeed, our analysis showed that *FOXM1* is highly expressed in multiple cancers (Figure 1) and associated with bad prognosis in breast cancer (Table 1 and Figure S2). These findings further reinforce previous studies reporting highly abnormal expression of FOXM1 in vast number of cancers and its correlation with poor prognosis [92, 94-97].

FOXM1 is known to regulate the expression of important AOS genes including *catalase*, *superoxide dismutase 2* (*SOD2*) and *PRDX3* [97, 98] which we found to be highly overexpressed in multiple cancers (group 6) (Table S1), at the exception of *catalase*, exclusively overexpressed in hepatocellular carcinoma (group 3) (Table S1). Like NRF2 [99], FOXM1 is induced by active RAS [97] and required for mutant RAS-mediated invasion, anchorage independent growth [100], and development of lung abnormalities *in vivo* [101].

FOXM1 can be inhibited by classic proteasome inhibitors [96, 102, 103], by piperlongumine that acts as a proteasome inhibitor [104] and promotes autophagic cell death [11], by a peptide derived from ARF [105] and by the CDK4/6 inhibitor PD0332991 [106]. Interestingly, PD0332991 is currently tested in clinical trials (phase II) in breast cancer patients emphasizing the importance of FOXM1 in breast cancer (for review see [95]). Because proteasome inhibitors are already used in the clinic to treat multiple myeloma [107, 108], it is possible that these inhibitors might prove being beneficial in breast cancers patients, whose tumors highly express FOXM1. Consistently, several ROS inducers effectively killed breast cancer cells when combined with proteasome inhibitors or siRNA-mediated knockdown of FOXM1 [103].

#### NF-kappaB

Our analysis revealed that among the cancer oxidative stress response genes identified (group 6), a number of them are NF-kappaB targets (Table 1). NF-kappaB is essential for proliferation, cell adhesion, inflammatory response and AOS response [109, 110], and its activity is deregulated in cancers [111]. Interestingly, a number of oxidative stress response genes are transcriptionally controlled by NF-kappaB including *SOD1*, *SOD2*, *GPX1*, *GSTP1* and the NRF2 targets *GCLC*, *GCLM*, *NQO1* and *HO-1* [112, 113]. This transcriptional regulation forms the basis for the protective role of NF-kappaB under oxidative stress [112]. This is especially relevant in the tumor context as we found that a number of these NF-kappaB targets are highly upregulated in multiple cancers (group 6, Table 1), supporting the notion of an elevated NF-kappaB activity in cancers as a strategy to manage oxidative stress conditions.

Several targeting approaches are being developed to inhibit NF-kappaB activity in cancers. The current strategy is to block NF-kappaB to sensitize tumors to chemotherapy and radiotherapy, since previous reports showed that inhibiting NF-kappaB leads to radiosensitization in radioresistant cancer cells [114, 115]. This is in agreement with the capacity of NF-kappaB to support an antioxidant program of which tumor cells may take advantage to resist oxidative stress-inducing therapies [116]. Thus, few natural compounds, such as curcumin, resveratrol and genistein, have been shown to inhibit NF-kappaB and to enhance the response to chemotherapeutic agents (for review[117]). A specific inhibitor of NF-kappaB nuclear translocation, namely dehydroxy-methylepoxy-quinomicin (DHMEQ), was shown to increase antitumor activities of taxane in a mouse model of thyroid cancer [118]. In addition, NF-kappaB activity can be blocked by direct inhibition of its upstream activator IKK, and the IKK inhibitor Bay 11-7082 leads to enhanced efficacy of cisplatin or paclitaxel in an ovarian tumor model [119, 120].

### The cancer oxidative stress response metabolic program: NADPH is a key factor

Our analysis showed that the TXN and glutathione pathways are up-regulated in multiple cancers at the transcriptional level and that high expression of a significant number of these genes is correlated with poor survival. Because both of these pathways rely on NADPH (Figure 3) it raises the possibility that cancer cells will be highly sensitive to NADPH depletion. Indeed, it was demonstrated that the survival of cancer cells requires activation of the AMPK pathway to maintain NADPH levels under metabolic stress, which is usually encountered within solid tumors [7, 121, 122]. Similarly, it was demonstrated that survival of cells under detachment conditions, a hallmark of transformation, is dependent on the pentose phosphate pathway that generates NADPH [10, 123]. Moreover, NRF2 was shown to promote cancer cell proliferation by increasing NADPH generation through transcriptional up-regulation of a number of enzyme-encoding genes including *G6PD* [74] (Figure 3). Another study showed that TAp73, a transcription factor that is a member of the p53 family [124-128], facilitates the growth of transformed and of cancer cells *in vitro* and *in vivo* by up regulating the expression of *G6PD* and therefore NADPH levels [129].

In our analysis, G6PD was found to be up-regulated in cancer (Figure 1) and bad prognosis in breast cancer (Table 1 and Figure S3). As *G6PD* fuels the TXN and glutathione pathways with NADPH (Figure 3), we speculate that G6PD might represent a highly attractive novel drug target. It is therefore encouraging that compounds that inhibit G6PD *in vitro* were synthesized recently [130]. However, more work is needed in order to find lead G6PD inhibitors as candidate anti-cancer drugs.

## CONCLUSIONS AND FUTURE PERSPECTIVES

The concept that the AOS response is utilized by cancer cells to promote their proliferation, adaptation, and resistance is now widely accepted by the scientific community and, therefore, numerous attempts to target AOS response genes as a therapeutic approach have been reported [10, 116, 131]. However, targeting endogenous proteins raises the concern of adverse off-target effects. Thus it is required to determine which proteins play a critical role in cancer as compared with normal tissues, as these are expected to offer a sufficient therapeutic window for intervention. Owing to the increasing availability of patient-derived gene expression, mutation, epigenetic, and survival data, it is now possible to use bioinformatics tools to screen for such targets in large cohorts for individual cancer entities as well as across histological entities [132].

Here, we used publicly available patient-derived gene expression and survival data, and identified genes that belong to two major detoxification pathways. Specifically, we show that genes belonging to the glutathione and TXN pathways are highly overexpressed in multiple cancers versus normal tissues and demonstrate that their high expression correlates with worse patient survival, pointing to a possible role of these genes as drug targets. Moreover, transcription factors such as NRF2, FOXM1, and NF-kappaB as well as key metabolic enzymes such as G6PD that altogether drive the activity of these pathways, were identified in our analysis providing further support to the argument that these are important drug targets. Because the TXN and glutathione pathways are hyperactive in multiple cancers, we hypothesize that simultaneous inhibition of both pathways via targeting common regulators such as NRF2 or common metabolic requirements such as NADPH, may be highly efficient and should be prioritized in drug development.

## Supplementary Figures and Tables




