# Supplementary material for: Anti-oxidative stress response genes: bioinformatic analysis of their expression and relevance in multiple cancers
Source: Oncotarget. 2013 Dec 15;4(12):2577–90. doi: 10.18632/oncotarget.1658 (PMC3926850; doi:10.18632/oncotarget.1658)
Supplement: Supplementary file 1 [file oncotarget-04-2577-s001.pdf]

# Anti-oxidative stress response genes: bioinformatic analysis of their expression and relevance in multiple cancers - Rotblat et al

Col1A1
Lung cancer GSE11969
p=0.000675

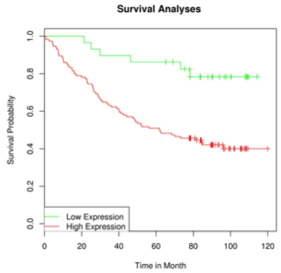


GAPDH
Lung cancer GSE11969
p=0.00185

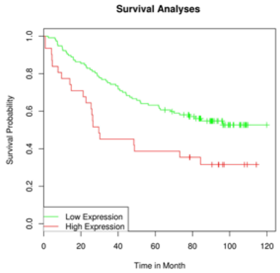


STK24
Lung cancer GSE11969
p=0.00169

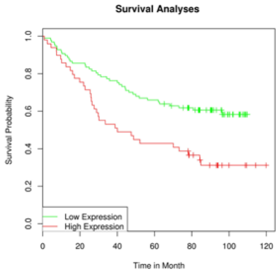


TXNRD1
Lung cancer GSE11969
p=0.00284

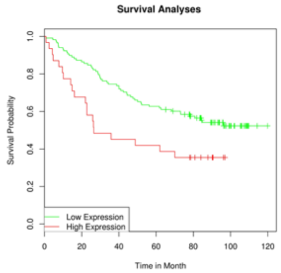


GCLC
Lung cancer GSE11969
p=0.00345

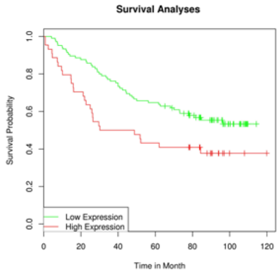


RNF7
Lung cancer GSE11969
p=0.00439

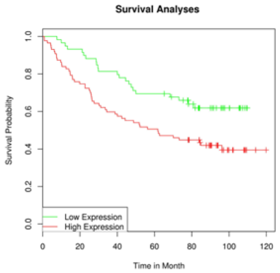


NQO1
Lung cancer GSE11969
p=0.0045

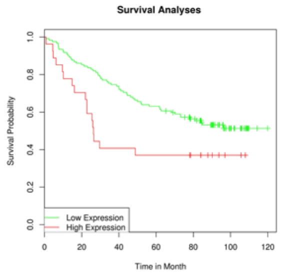


TXN
Lung cancer GSE11969
p=0.00789

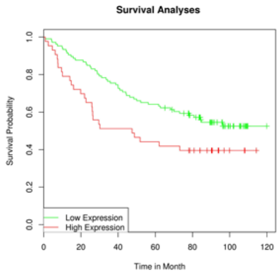


GSS
Lung cancer GSE11969
p=0.009934

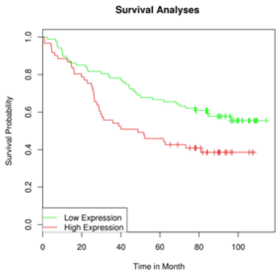


NFKB
Lung cancer GSE11969
p=0.00849

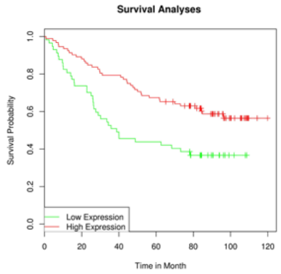


**Figure S1: Kaplan-Meier plots for all oxidative stress response genes found to be highly expressed in cancers whose expression significantly correlates with survival in lung cancer.**

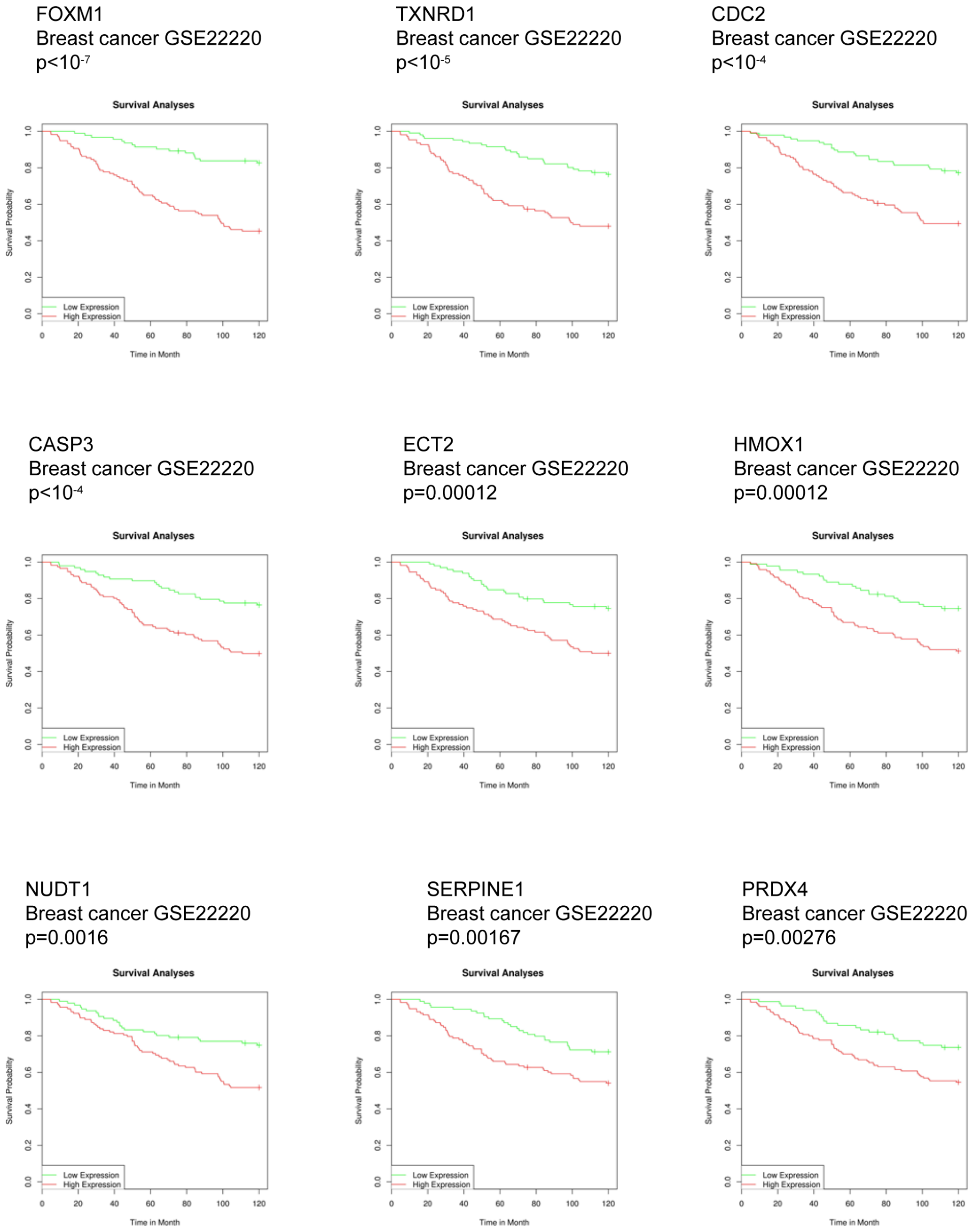


**Figure S2-S3: Kaplan-Meier plots for all oxidative stress response genes found to be highly expressed in cancers whose expression significantly correlates with survival in breast cancer.**

LONP1
Breast cancer GSE22220
p=0.0031

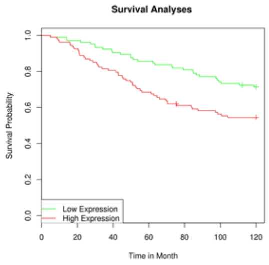


GAPDH
Breast cancer GSE22220
p=0.00321

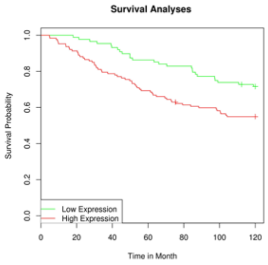


SNRX1
Breast cancer GSE22220
p=0.00321

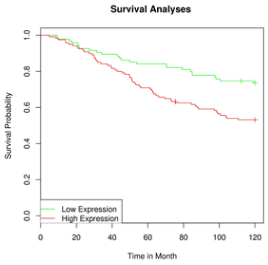


PSNB5
Breast cancer GSE22220
p=0.0037

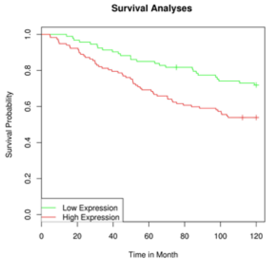


BTG3
Breast cancer GSE22220
p=0.00357

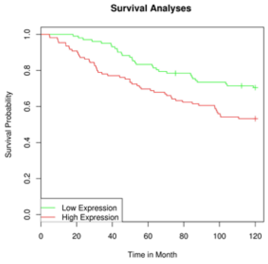


EGLN1
Breast cancer GSE22220
p=0.00586

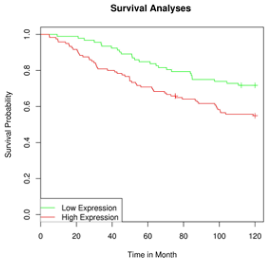


G6PD
Breast cancer GSE22220
p=0.00748

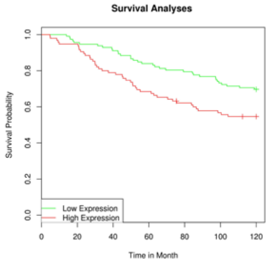


SELS
Breast cancer GSE22220
p=0.00844

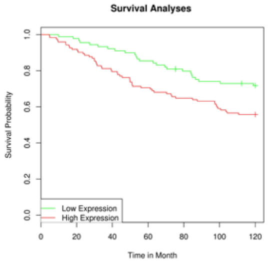


PON2
Breast cancer GSE22220
p=0.00457

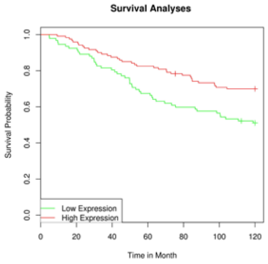


SIRT1
Breast cancer GSE22220
p=0.00918

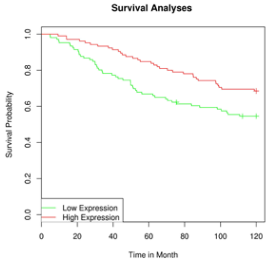


**Figure S2-S3: Kaplan-Meier plots for all oxidative stress response genes found to be highly expressed in cancers whose expression significantly correlates with survival in breast cancer.**
